# An Integrated Glycosylation Signature of Rheumatoid Arthritis

**DOI:** 10.3390/biom13071106

**Published:** 2023-07-12

**Authors:** Oleg A. Mayboroda, Guinevere S. M. Lageveen-Kammeijer, Manfred Wuhrer, Radboud J. E. M. Dolhain

**Affiliations:** 1 Center for Proteomics and Metabolomics, Leiden University Medical Center, 2333 ZA Leiden, The Netherlands; o.a.mayboroda@lumc.nl (O.A.M.); g.s.m.kammeijer@rug.nl (G.S.M.L.-K.); 2 Analytical Biochemistry, University of Groningen, 9700 RB Groningen, The Netherlands; 3 Department of Rheumatology, Erasmus Medical Center, 3015 CN Rotterdam, The Netherlands

**Keywords:** glycosylation, rheumatoid arthritis, antibody

## Abstract

Rheumatoid arthritis (RA) Is a highly prevalent autoimmune disease that affects the joints but also various other organs. The disease is characterized by autoantibodies that are often already observed pre-disease. Since the 1980s, it has been known that antibody glycosylation is different in RA as compared to control individuals. While the literature on glycosylation changes in RA is dominated by reports on serum or plasma immunoglobulin G (IgG), our recent studies have indicated that the glycosylation changes observed for immunoglobulin A (IgA) and total serum *N*-glycome (TSNG) may be similarly prominent, and useful in differentiating between the RA patients and controls, or as a proxy of the disease activity. In this study, we integrated and compared the RA glycosylation signatures of IgG, IgA and TSNG, all determined in the pregnancy-induced amelioration of rheumatoid arthritis (PARA) cohort. We assessed the association of the altered glycosylation patterns with the disease, autoantibody positivity and disease activity. Our analyses indicated a common, composite glycosylation signature of RA that was independent of the autoantibody status.

## 1. Introduction

Rheumatoid arthritis (RA) is a highly prevalent autoimmune disease that affects the joints but also various other organs [1]. The disease is characterized by autoantibodies that are often already observed pre-disease [2]. Autoreactive B cells and autoantibodies are considered to contribute to the development of RA [3]. Furthermore, the T regulatory cells (Tregs) have a significant impact on the pathogenesis of RA; the primary function of the Tregs is to suppress the activity and function of the other immune cells [4]. The current models of the Tregs role in RA explain a decline of their suppressive function by a conversion into pro-inflammatory effector T cells. This phenotypic conversion is triggered by the cytokine environment and availability of interleukin-2 (IL-2) in the first place [5].

Since the 1980s, it has been known that antibody glycosylation is different in RA as compared to control individuals [6,7,8,9]. Specifically, the galactosylation and sialylation of immunoglobulin G (IgG) Fc *N*-glycans attached to the conserved *N*-glycosylation site of the CH2 domain is low in RA patients [10]. The extent of hypogalactosylation has been shown to reflect the disease activity [11]. Not only the total or bulk IgG show the low galactosylation phenotype, but also the RA-associated autoantibodies such as the anti-citrullinated peptide antigen (ACPA) IgG autoantibodies, both from the circulation and from the synovial fluid of inflamed joints [12]. While IgG Fc glycosylation is known to influence the IgG effector functions in various ways, and IgG glycosylation has been shown to affect the pathogenicity of autoantibodies in various murine autoimmune disease models, the potential pathogenic role of IgG Fc glycosylation in RA is still subject to debate [13].

Next to IgG, other proteins in the circulation have shown altered glycosylation profiles in RA. This includes immunoglobulin A (IgA) and alpha-1-antitrypsin (AAT) [6,14,15]. Consequently, the total *N*-glycome enzymatically released from the pool of the serum glycoproteins has been shown to be altered in RA, reflecting the altered *N*-glycosylation of IgG and IgA, but also other serum proteins [16].

Whilst altered *N*-glycosylation in RA has been broadly assessed for serum and plasma proteins, the analysis of IgA modifications in RA revealed a highly skewed *O*-glycome in the hinge region of IgA [17].

While the literature on glycosylation changes in RA is dominated by reports on serum or plasma IgG, our recent studies have indicated that the glycosylation changes observed for IgA and total serum *N*-glycome (TSNG) may be similarly prominent, and useful in differentiating between RA patients and controls, or as a proxy of the disease activity [14,18,19,20]. In this study, we integrated and compared the RA glycosylation signatures of IgG, IgA and TSNG, all determined in the pregnancy-induced amelioration of rheumatoid arthritis (PARA) cohort. We assessed the association of the altered glycosylation patterns with the disease, autoantibody positivity and disease activity. Our analyses indicated a common, composite glycosylation signature of RA that was independent of the autoantibody status.

## 2. Materials and Methods

Data collection and analysis. Data from three previously published studies on the PARA cohort were used for this report: Bondt et al. 2013 for IgG block [20], Bondt et al. 2017 for IgA [19] and Reiding et al. 2018 for TSNG [18]. All data analysis was performed with the R statistical environment (http://www.r-project.org/, R versions 4.2.2, 4.2.1). Basic data table handling was performed with the help of the tidyverse package (version 1.3.2). Normalization function of the clusterSim (version 0.48-1) package was used for the data normalization prior to modeling. Missing data was imputed using a multivariate imputations by chained equations (MICE) algorithm, which enables imputation of each incomplete variable by a separate model (R package mice 3.12.0). The method is an iterative procedure which specifies the multivariate imputation model on a variable-by-variable basis. To dissect an optimal subset of glycans, we used a regression approach, namely, the DIABLO (Data Integration Analysis for Biomarker discovery using Latent variable approaches for ‘Omics studies) tool of the mixOmics package (version 6.20.0) [21,22]. DIABLO combines a surprised multiblock modeling with variable selection. For calculation of the logistic regression metrics, the following packages were used: car 3.0, caret 6.0, broom 0.7.10. and lsmeans 2.3. Logistic regression model optimization was performed using the regsubset function of the leaps package (version 3.1) followed by a manual curation for collinearities. For data visualization the ggplot2 package (version 3.3.6) was used.

## 3. Results

### 3.1. Data Set Overview

Three previously acquired glycomics data sets available for the PARA study [18,19,20]—a nationwide prospective cohort study on pregnancy and RA, were integrated and explored. Serum samples of 250 RA patients were collected in the Netherlands between 2002 and 2009. The reference group consisted of 32 healthy participants [23]. For the analyses in the current study, only a single, six months postpartum time point was used (243 RA patients and 31 healthy participants). Unlike the already published reports on the glycoprofiles in the PARA cohort, the analysis was built not on the calculated, composite values (the derived glycosylation traits) but on the relative abundances of the individual (direct) glycan traits. The glycan traits were screened for missing values and the ones with more than 25% of the missing values were excluded from further analysis. The remaining features were imputed if necessary. The final data set contained 182 variables belonging to three experimental modalities: IgG Fc *N*-glycans (50 variables: IgG1 and IgG2/3 20 variables each and IgG4 10 variables), IgA *O*-glycans (53 variables) and TSNG—total serum *N*-glycans (78 variables) (Supplementary Table S1). Supplementary Figure S1 summarizes the patient selection, data modalities and outlines the general analysis strategy.

### 3.2. Univariate Analysis of the Individual Datasets

**Figure 1:**
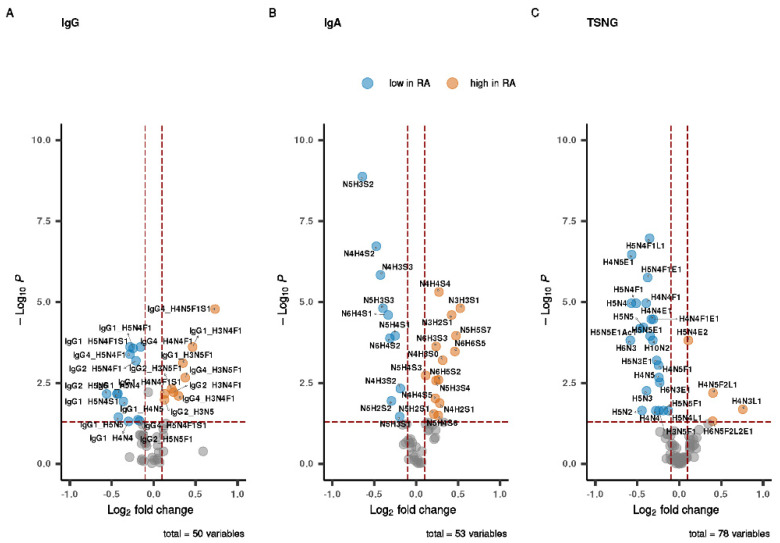
Volcano plots of differences in glycosylation between RA patients and controls. For the three selected data modalities (**A**) IgG *N*-glycans, (**B**) IgA *O*-glycans and (**C**) TSNG of RA patients and controls at postpartum timepoint, a comparison was made of relative abundances of each glycosylation trait using a nonparametric univariate test, the *p*-values were corrected for multiple testing. All significantly different structures are colored. All IgG subclasses showed differences in Fc glycosylation profiles between RA and controls. Consistent with the literature, non-galactosylated glycoforms were lower in RA. Interestingly, a number of monogalactosylated glycoforms such as IgG1_H4N4F1 and IgG4_H4N5F1S1 were found to be lower in RA. All measured values are summarized per the dataset in Supplementary Table S1.

To provide an overview of the differential abundance for the glycosylation traits between the RA patients and the healthy participants, we performed a univariate analysis on each of the three datasets. The data are summarized in Figure 1 and Supplementary Table S1. We found that each data set contained a number of the significantly altered traits.

For IgG, all three subclasses showed altered Fc glycosylation in RA. Glycoforms with low levels of galactosylation, as indicated by a content of three or four hexoses (H), were increased in RA (Figure 1A, Supplementary Table S1). On the other hand, glycoforms that were digalactosylated (H5) and sialylated (S) were low in RA. Intriguingly, a sialylated glycoform of IgG4 (IgG4_H4N5F1S1) did not follow this pattern, as it was found to be prominently increased in RA. In addition, despite a high overall Fc *N*-glycan fucosylation level in RA, the fucosylated glycoforms of IgG1 (IgG1 H5N4F1S1, IgG1 H5N4F1) were found to be lower in RA.

For IgA *O*-glycosylation, the pattern of the glycoforms that were found to be elevated or decreased in RA was very complex. Overall, the glycoforms with very high levels of galactosylation (5 or 6 hexoses) and the glycoforms with high levels of sialylation (4–7 sialic acids) were largely decreased in RA (Figure 1B, Supplementary Table S1). For the glycoforms with lower numbers of hexoses and sialic acids, the picture was more diverse, with some of them being elevated in RA, and others decreased. Finally, some differences were found in the distribution of the significant traits between the datasets. For example, while in the IgG and IgA datasets the distribution appeared as more or less similar, in the TSNG it was “skewed” and the majority of the significant structures were lower in RA (Figure 1C, Supplementary Table S1).

### 3.3. Integrative Analysis of the Glycomic Datasets

In order to establish an integrated glycosylation signature of rheumatoid arthritis, we analyzed the data presented in Figure 1 using a multiblock regression approach, namely the DIABLO (Data Integration Analysis for Biomarker discovery using Latent variable approaches for ‘Omics studies) tool [21]. Supplementary Figure S2 shows the signature in the form of a clustered image map. Remarkably, all the structures contributing to the signature were considered to be rather complex, consisting either of at least four hexoses or the structures including fucosylation and a high level of sialylation. Except for a single non fucosylated feature in the IgG Fc *N*-glycans block (IgG1 H5N4), TSNG was the only data set from which simpler structures lacking sialylation or fucosylation were included in the signature.

**Figure 2:**
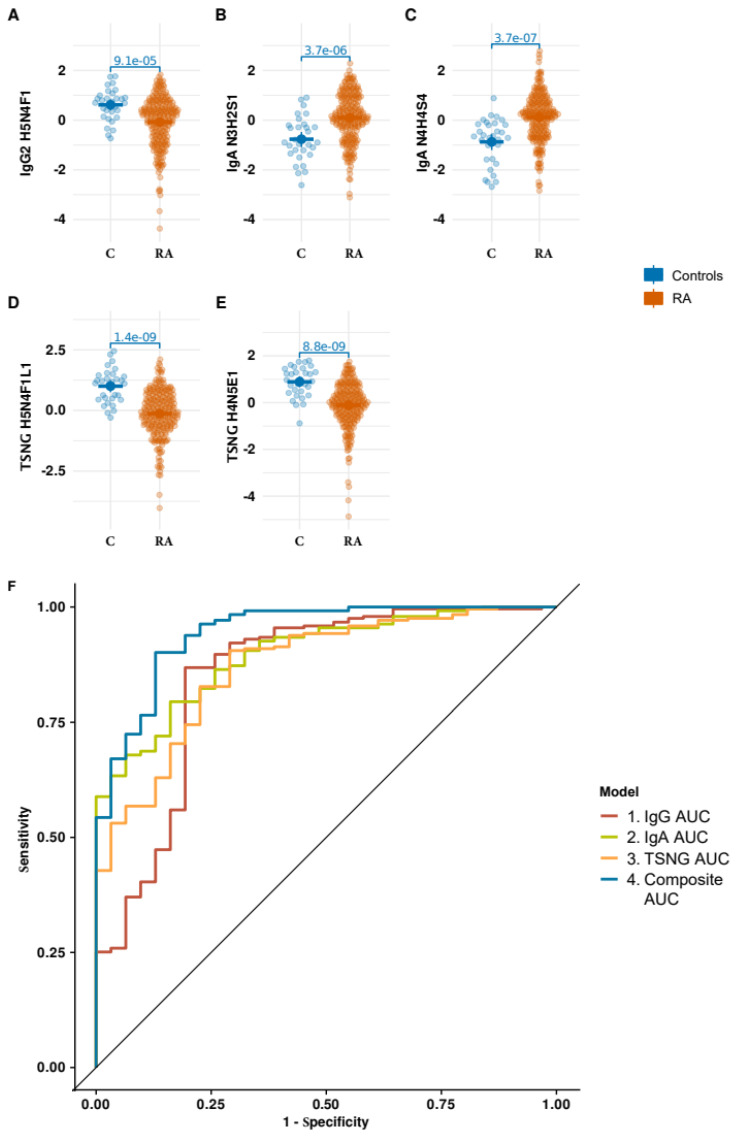
Optimized composite glyco-trait signature of RA outperforms the individual ones. (**A**–**E**) The composite signature (29 glycans, Supplementary Figure S2) was optimized to comply with the assumptions of the logistic regression models. The optimal model consisted of five predictors. (**F**) A comparison of the ROC curves for the composite and the individual models. To compare the composite models with the models built on each individual data modality, we built an optimized logistic regression model for IgG, IgA and TSNG separately. The composite model clearly outperformed the others on basis of AUC. Supplementary Table S2 the summarizes all model metrics.

The complex signature presented on the Supplementary Figure S2 gives an overview of the glyco-traits associated with RA, but using such a complex combination of the measured glyco-traits for a prediction, etc., would be unpractical. Therefore, as a next step, we decided to adopt the signature for a logistic regression. For the given data, the most important part of the procedure was to remove the possible multicollinearity effects which could inflate the resulting logistic regression models and result in overoptimistic performance metrics. Figure 2A–E shows the “beeswarm” plots for the optimized selection of glyco-traits consisting of five structures: one from the IgG Fc *N*-glycans data set (IgG2/3-H5N4F1), two from TSNG (H5N4F1L1 and H4N5E1) and two from IgA O-glycans (N3H2S1 and N4H4S4). Furthermore we compared the composite/integrative model with the models built for each individual data set. A model optimization was performed to find an optimal predictor combination for IgG Fc *N*-glycans, IgA and TSNG separately. Figure 2F shows the receiver operating characteristic (ROC) plots for all the models. The area-under-the-curve (AUC) of the composite model was found to be 0.945. The IgA *O*-glycans data set showed the highest AUC (0.896) among the models built on the individual data sets, with TSNG and IgG showing values of 0.871 and 0.852, respectively. Supplementary Table S2 summarizes all the essential metrics (odds ratios, standardized β coefficients) for the integrative and individual models.

Finally, our data set had an important weakness due to the seven-and-a-half-times difference in the numbers of the patients and controls. This effect, known as a class imbalance, leads to a overestimation of the resulting model performance because the model tends to “favor” the prevalent class. To address this, we performed data re-sampling using the random over-sampling examples (ROSE) method based on a smart bootstrapping of the original data [24]. Supplementary Figure S3 shows the ROC curves for the composite signature modeled using the original data and the re-sampled data with balanced classes. As expected, the balanced model had lower AUC (0.881 for the balanced model versus 0.945 for the original one).

### 3.4. Performance of the Optimized Signature on ACPA Negative Strata of the Data

**Figure 3:**
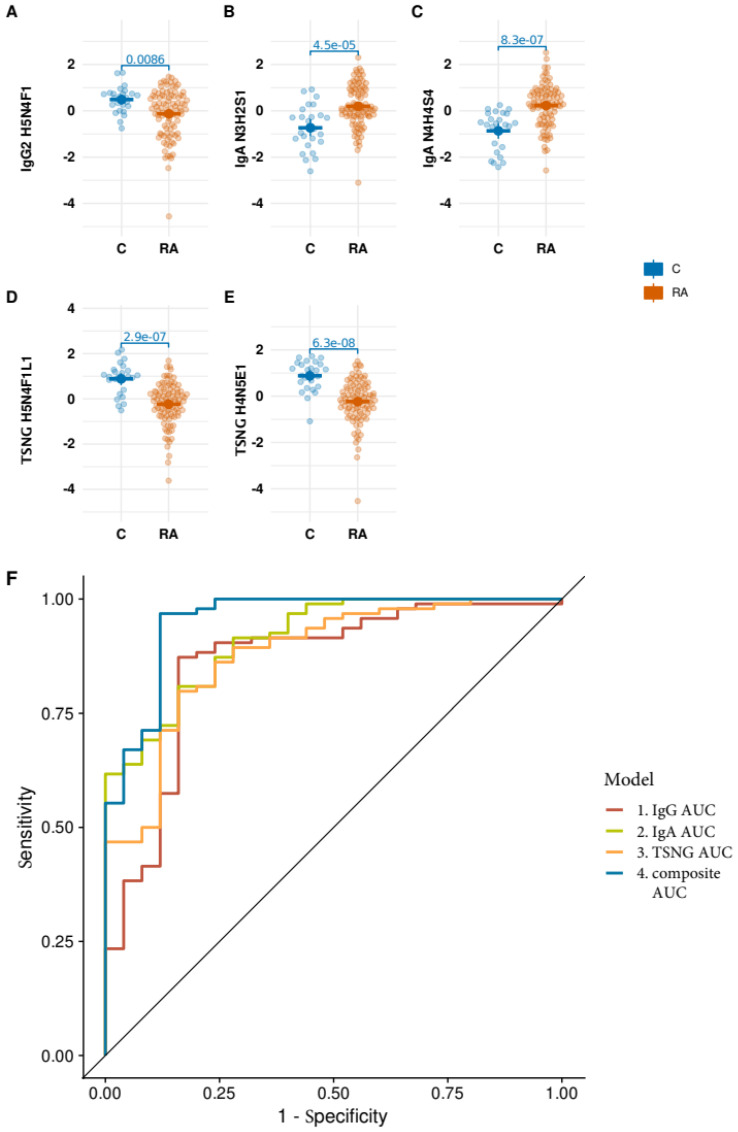
Optimized glycan signature performance on ACPA negative strata. To evaluate the signature performance on the ACPA-negative part of the data we built the logistic regression models only on this part of the data. The signature performance on ACPA-negative strata was similar to the entire data set (**A**–**E**). The AUC of the composite model was 0.954 (balanced model: 0.881). The IgA *O*-glycan data set showed the highest AUC (0.808) among the models built on the individual data sets, with TSNG and IgG showing the values 0.786 and 0.726, respectively (**F**).

One of the notable features of the PARA study was that it included cases both with and without the presence of antibodies to citrullinated proteins (ACPA). Citrullination is a post-translational modification process in which the amino acid arginine is converted to citrulline by the enzyme peptidylarginine deiminase (PAD) [25]. ACPA-negative RA, which is sometimes referred to as a seronegative form of the disease, accounts for approximately 40% of all RA cases [26]. The available clinical and experimental data show that ACPA-positive and ACPA-negative forms are the distinct subtypes of RA [27]. While the ACPA-positive form is considered as being more severe and implies a higher risk of developing complications (e.g., heart disease), the ACPA-negative form could be more “evasive” as one of the important diagnostic features, namely antibodies to citrullinated proteins, is missing. To this end, we tested the performance of the optimized selection of glyco-traits on the ACPA-negative stratum of the cohort. Figure 3 shows that AUC of the composite model built on the ACPA-negative stratum was 0.954 (for the balanced model: 0.881). The IgA O-glycan data set showed the highest AUC (0.808) among the models built on the individual data sets, with TSNG and IgG showing the values of 0.786 and 0.726, respectively. Thus, the model built on the optimized selection of the glyco-traits showed no drop of performance on the ACPA-negative stratum. Moreover, we compared the relative abundances of the glyco-traits between the positive and negative strata. Supplementary Figure S4 shows that none of the five selected predictors were significantly different between the two groups.

### 3.5. Association of the Individual Glyco-Traits with DAS28

**Figure 4:**
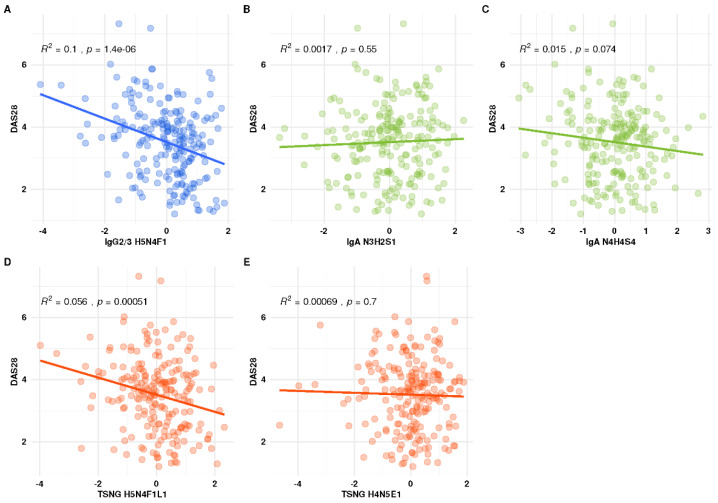
Linear associations between DAS28 and the predictors (glyco-traits) of the composite model. A linear model with DAS28 as a response variable was built for each of the selected glyco-traits. Only two features show a significant association with DAS28, being IgG2 H5N4F1 (*p* = 1.39 × 10^−6^) and H5N4F1L1 (*p* = 0.0005). The variance explained by the model was only 10% for IgG2 H5N4F1 and 5% for TSNG H5N4F1L1.

The biological origin of the glyco-traits data used for this report, namely IgG, IgA and the serum proteins (TSNG) implied that the selected features could reflect not only an actual status of the disease but also the degree of inflammation. To explore this possible dependency, we used linear associations between the DAS28 scores (disease activity score with 28-joint count) and the traits of the glycan signature. In the PARA study, we used the DAS28-CRP-3 variant of the DAS28 score, which was a composite joint score based on 28 swollen and tender joints and C-reactive protein (CRP). Figure 4 shows that of the five glyco-traits, only the two fucosylated species, namely IgG2/3 H5N4F1 and TSNG H5N4F1L1 demonstrated a statistically significant association with DAS28-CRP-3. We hypothesized that with a blood test of CRP, produced by the liver in response to inflammation, being a part of the score, this score of disease activity was also related to the inflammation. Thus, our data showed that while we could not exclude a contribution of inflammation to the resulting signature, it was not the main factor.

## 4. Discussion

Over several decades, the idea that the changes in serum protein glycosylation patterns could serve as the markers of a disease onset or progression has been gaining strong experimental and clinical support. According to a recent report by Dotz et al. [28], a PubMed search query for ‘glycan AND biomarkers’ returned roughly 10,000 items only for a period between 2009 and 2018. In cancer, changes in the glycosylation patterns of serum proteins were found to correlate with the tumor stage [29]. Autoimmune diseases and RA in particular are probably the most extensively studied clinical entities with regards to the alterations of IgG glycosylation. A seminal manuscript of Parekh et al. published in 1985 demonstrated that a “pattern” of IgG galactosylation of RA patients is “shifted” towards lower galactosylation in serum [7]. By now, IgG galactosylation patterns have been shown to be a good indicator of the disease activity [16]. In the same manuscript, the authors postulated that the disease-related alterations of glycosylation are not restricted to IgG only, but may also be found on other serum glycoproteins. Accordingly, studies of the *N*-glycome of enzymatically-released from the total serum glycoproteins suggest that RA-associated glycosylation changes are not restricted to IgG and IgA, but may affect the acute-phase proteins such as alpha-1-antitrypsin [30]. To obtain a thorough assessment of glycosylation on various protein targets (e.g., IgG and IgA), focused workflows should be applied that include a protein enrichment step prior to glycans release or proteolytic digestion. However, this approach leads to fragmented data reporting, where each data block is reported and interpreted separately [18,19,20]. 

In this manuscript, we presented an integrative, post-acquisition analysis of three data sets from the PARA study. We based our analysis on the relative abundances of individual glyco-traits, rather than the calculated composite values (the derived glycosylation traits). Utilizing a matrix-based method for integrative analysis [22], we dissected a complex descriptive signature consisting of 29 glyco-traits. The signature reflected practically all the established alterations of the glycosylation, e.g., reduced galactosylation, sialylation and the changes in fucosylation pattern. An optimized signature, tailored to a more convenient reporting method, namely logistic regression, included glyco-traits from all three datasets and outperformed all the signatures built on the individual datasets. Of the individual datablocks, the IgA *O*-glycans dataset showed the best performance. To this end, we can conclude that by combining data from multiple glyco-workflows, one could obtain a good predictive model for RA diagnosis. Yet, an obvious weakness of such an integrative solution is that the data production requires a combination of the labor-intensive analytical workflow. A possible compromise solution could be to use the best performing individual layer, which was the IgA glycosylation data. Yet, development of an analytical solution with a better coverage is desirable. Finally, while the three underlying studies concentrated on discovering and determining the most effective predictive combinations of variables, there is still a lack of (patho-)physiological understanding that may shed light on the associations of glycosylation with inflammation and disease status. We demonstrated that, when using the DAS28 score as a measure of inflammation, only two of the five glyco-traits, IgG2/3 H5N4F1 and TSNG H5N4F1L1, exhibited a weak, yet statistically significant linear association with the score, indicating that the RA glycosylation signature could only for a minor part be attributed to the changes in DAS28 and inflammation.

To summarize, our study revealed the contribution of IgG and IgA glycosylation as well as TSNG to a blood glycosylation signature of RA. The fact that this glycosylation signature appeared to be equally valid for autoantibody-positive and -negative RA patients indicated some potential for this signature in the diagnosis of RA. This was further supported by the fact that the signature appeared to be largely independent of the disease activity. It has to be taken into account, however, that the PARA cohort was considerably homogeneous as it only covered young women. A further evaluation of the composite RA glycomic signature may therefore aim at replication in a broader, more heterogeneous cohort of RA patients. Likewise, the validity of the signature in early RA and its possible emergence in pre-RA conditions require further studies.

## Data Availability

The links to the original publications of the used data are provided in Supplementary Table S1.

## Supplementary Materials

The following supporting information can be downloaded at: https://www.mdpi.com/article/10.3390/biom13071106/s1. Supplementary Tables: Supplementary Table S1. A statistical summary of all three data modalities per group (RA and Controls): median and interquartile range—Median (IQR), mean and standard deviation—Mean (SD), range and *p*-value (MW-test). Supplementary Table S2. Summary of the logistic regression models optimized for the individual data modalities and the composite model. Supplementary Figures: Supplementary Figure S1. A general outline of the patient data, analytical data modalities and analysis strategy chosen of the current report. Supplementary Figure S2. Integrative analysis of the glycomic datasets. The heatmap represents a complex signature consisting of 29 glyco-traits from all three data modalities. The signature was generated using the data integration analysis for biomarker discovery using latent components (DIABLO) method. DIABLO is an integrative method that extracts common information across different data types and selects a subset of molecular features discriminating between study groups. The model was tuned for maximal class separation. The variable selection process was based on class identity, yet the clustering used for the visual enhancement was completely unbiased. Supplementary Figure S3. The ROC plots for the original compost model presented in the Figure 3 and a model corrected for the class imbalance issue. The AUC of the model built on the available data was 0.945, for the re-sampled model with the balanced classes the AUC was 0.881. Supplementary Figure S4. A comparison of the relative abundances between ACPA-positive and ACPA-negative strata for the glyco-traits contributing to the optimized signature. Neither of them showed a statistically significant difference.
