# Supplementary material for: An Integrated Glycosylation Signature of Rheumatoid Arthritis
Source: Biomolecules. 2023 Jul 12;13(7):1106. doi: 10.3390/biom13071106 (PMC10377307; doi:10.3390/biom13071106)
Supplement: Supplementary file 1 [file biomolecules-13-01106-s001.zip › Figure S1.pdf]

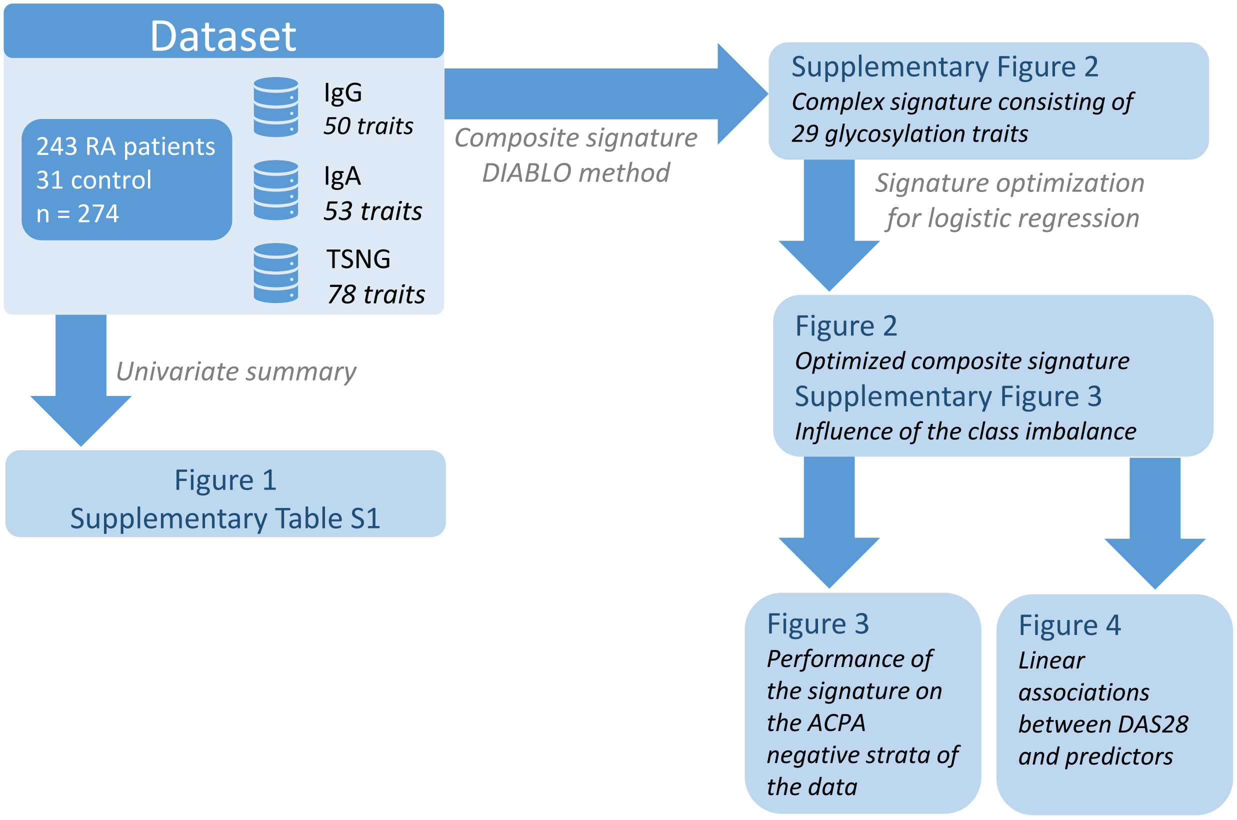
Dataset
243 RA patients
31 control
n = 274
IgG
50 traits
IgA
53 traits
TSNG
78 traits
Composite signature
DIABLO method
Supplementary Figure 2
Complex signature consisting of
29 glycosylation traits
Signature optimization
for logistic regression
Figure 2
Optimized composite signature
Supplementary Figure 3
Influence of the class imbalance
Univariate summary
Figure 1
Supplementary Table S1
Figure 3
Performance of the signature on the ACPA negative strata of the data
Figure 4
Linear associations between DAS28 and predictors
