# Supplementary material for: An Integrated Glycosylation Signature of Rheumatoid Arthritis
Source: Biomolecules. 2023 Jul 12;13(7):1106. doi: 10.3390/biom13071106 (PMC10377307; doi:10.3390/biom13071106)
Supplement: Supplementary file 1 [file biomolecules-13-01106-s001.zip › Table S2.pdf]

Supplementary Table S2. Summary of the logistic regression models

Variables	St. β (SE)	OR (95% CI)	AUC (95% CI)	Chi	Pr (>Chi)
**Models built on the individual datasets**					
**IgG**					
IgG4 H4N5F1S1	1.418 (0.350)	4.130 (2.201– 8.776)	0.852 (0.757-0.932)	55.786	4.666e-12
IgG1 H5N4F1S1	- 1.486 (0.347)	0.226 (0.108 – 0.425)			
IgG2 H4N4F1	-0.654(0.296)	0.520(0.282 - 0.902)			
**IgA**					
IgA N3H2S1	1.045 (0.245)	2.843 (1.797 – 4.729)	0.896 (0.845-0.942)	68.108	1.089e-14
IgA N5H5S7	1.371 (0.263)	3.940(2.449 – 6.913)			
IgA N6H3S3	1.035(0.264)	2.815(1.710 - 4.863)			
**TSNG**					
TSNG H5N4F1L1	-1.505 (0.350)	0.222 (0.106-0.425)	0.871 (0.805-0.927)	58.337	1.332e-12
TSNG H5N4F1	-0.639 (0.311)	0.527 (0.281-0.959)			
TSNG H4N5F2L1	0.632 (0.250)	1.882(1.166 -3.126)			
**Integrated model**					
IgG4 H5N4F1	-0.729 (0.393)	0.482 (0.209– 0.980)	0.945 (0.899 - 0.982)	108.677	7.790e-22
IgA N3H2S1	1.115(0.352)	3.050 (1.597 – 6.413)			
IgA N4H4S4	1.307 (0.329)	3.694 (2.032- 7.491)			
TSNG H5N4F1L1	-1.292(0.434)	0.275 (0.109 – 0.609)			
TSNG H4N5E1	-1.253 (0.446)	0.286 (0.110 - 0.641)			

AUC, area under the curve; CI, confidence interval; OR, odds ratio; St. β, standardized β;

Supplementary Table S2A

Model	Cross-validation accuracy of the models (CV Accuracy)	CV Accuracy SD
IgG	0.915	0.027
IgA	0.893	0.034
TSNG	0.896	0.032
Integrated	0.948	0.031
