# Supplementary figures and images for: An Integrated Glycosylation Signature of Rheumatoid Arthritis
Source: Biomolecules. 2023 Jul 12;13(7):1106. doi: 10.3390/biom13071106 (PMC10377307; doi:10.3390/biom13071106)

Color key

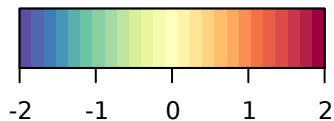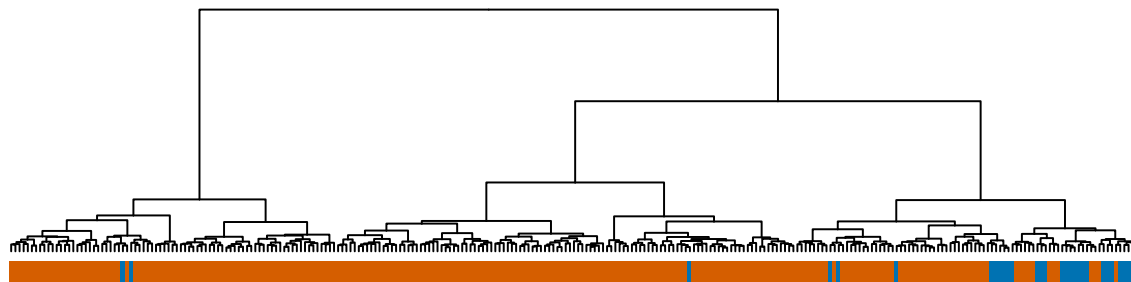

Rows - Data sets

- IgG
- IgA
- TSNG

Columns

- Controls
- RA

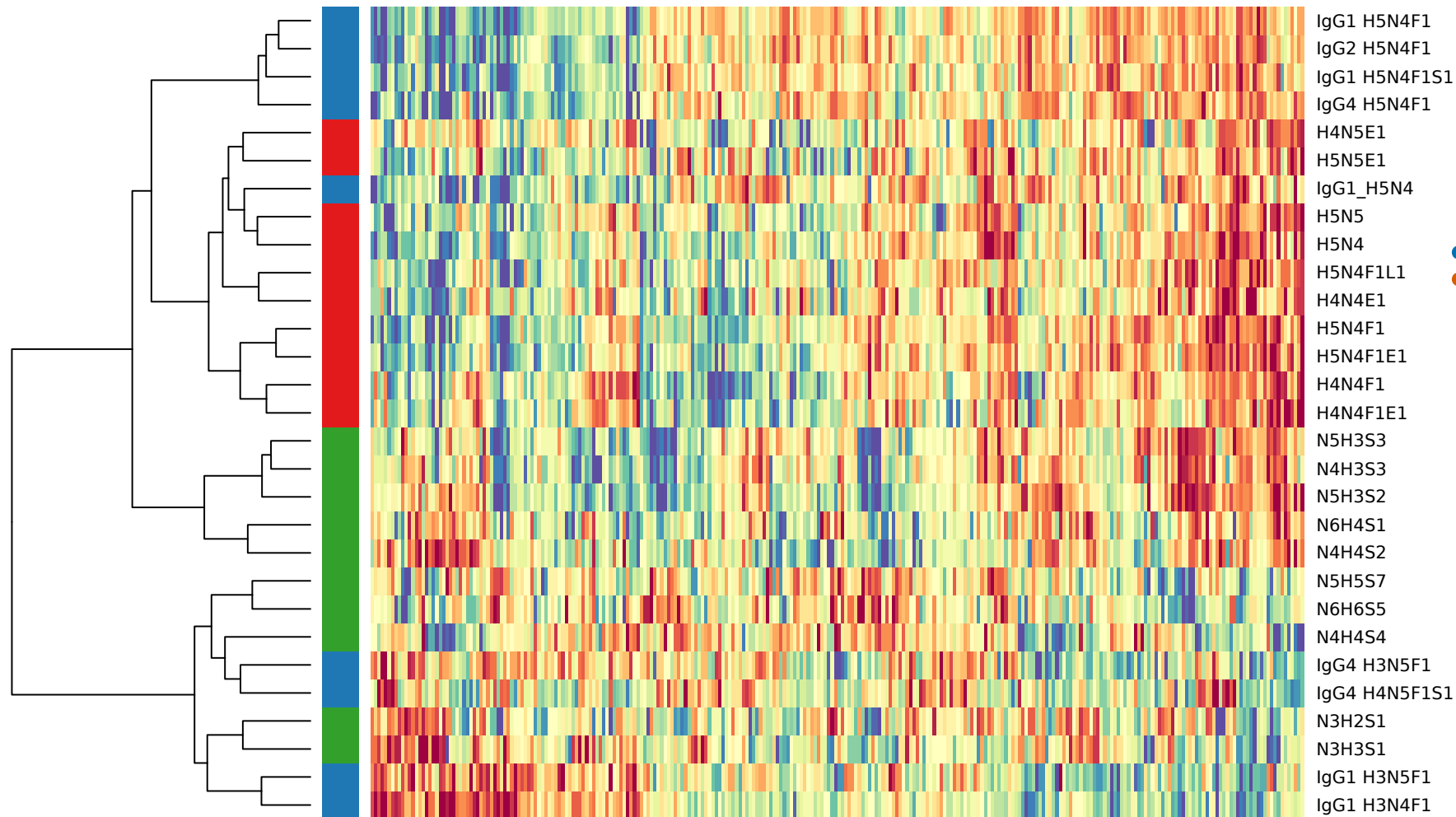

Supplement: Supplementary file 1 [file biomolecules-13-01106-s001.zip › Figure S2.pdf]

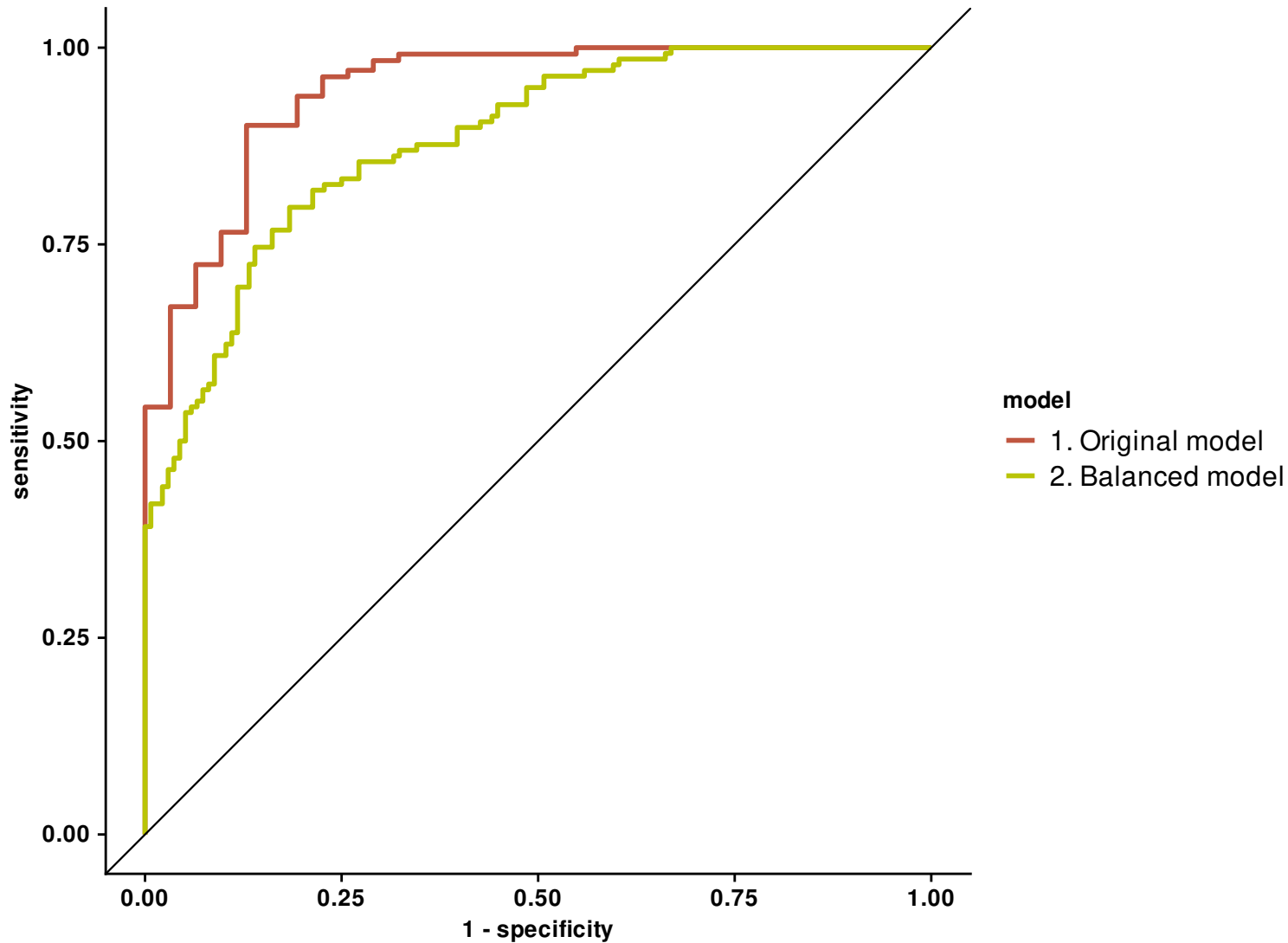

Supplement: Supplementary file 1 [file biomolecules-13-01106-s001.zip › Figure S3.pdf]

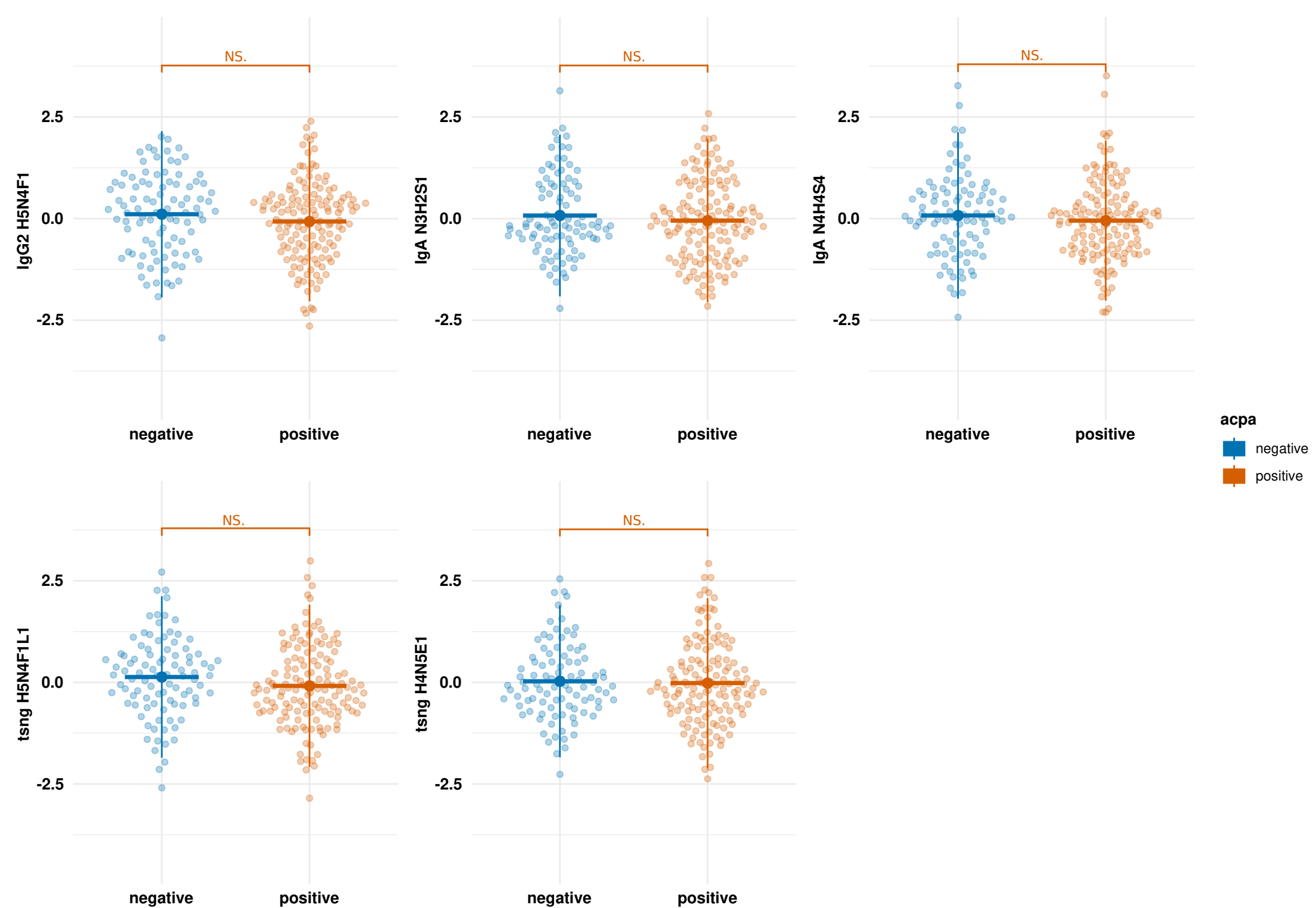

Supplement: Supplementary file 1 [file biomolecules-13-01106-s001.zip › Figure S4.pdf]
